# Supplementary material for: Comparisons of Nutrient Intakes and Diet Quality among Water-Based Beverage Consumers
Source: Nutrients. 2019 Feb 1;11(2):314. doi: 10.3390/nu11020314 (PMC6412440; doi:10.3390/nu11020314)
Supplement: Supplementary file 1 [file nutrients-11-00314-s001.pdf]

Supplemental Material

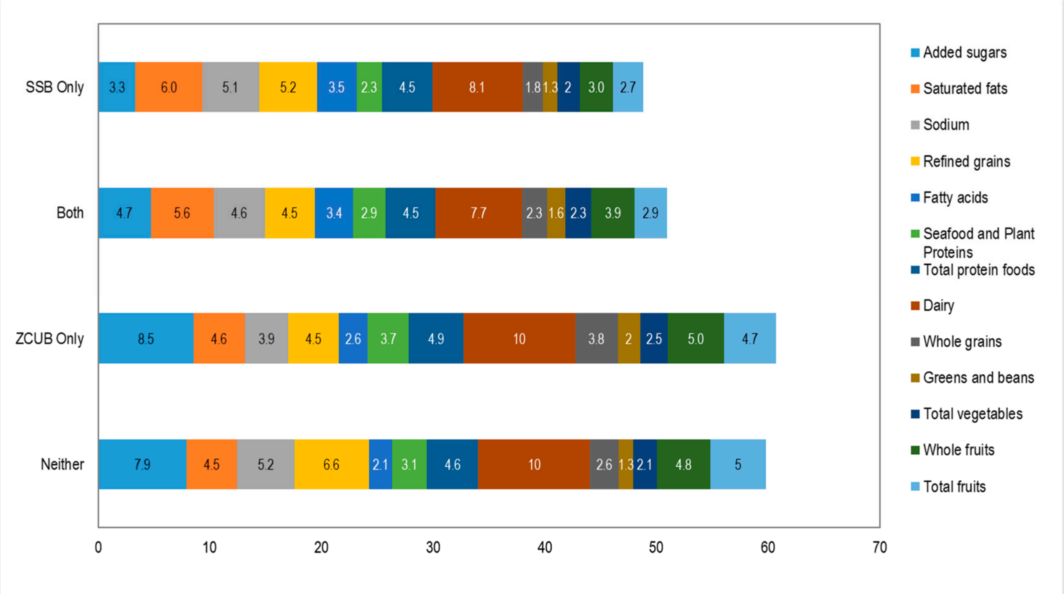

Figure S1. Mean scores for sub-components of the HEI-2015 among children 2-18 years by ZCUB consumer group, NHANES 2009-2016; HEI-2015 scores calculated using the population ratio method with Day 1 dietary intake.

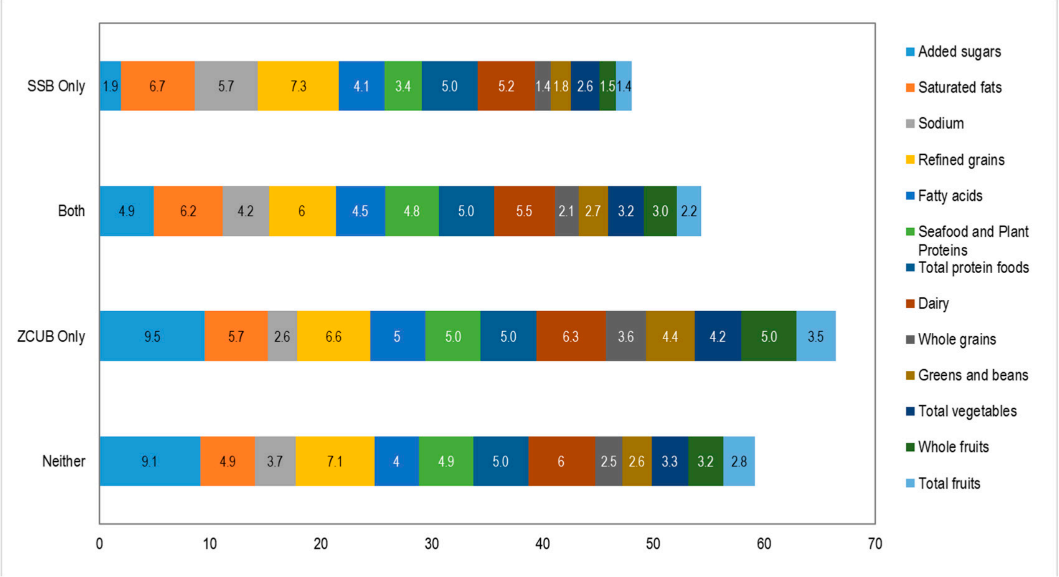

Figure S2. Mean scores for sub-components of the HEI-2015 among adults 19-64 years by ZCUB consumer group, NHANES 2009-2016; HEI-2015 scores calculated using the population ratio method with Day 1 dietary intake.

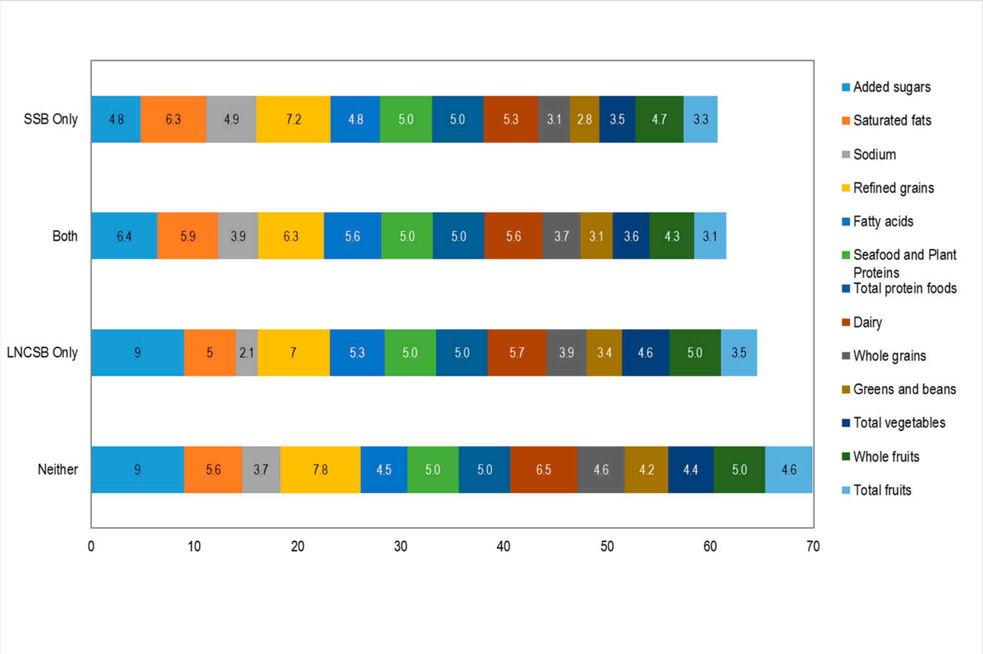

Figure S3. Mean scores for sub-components of the HEI-2015 among older adults 65+ years by ZCUB consumer group, NHANES 2009-2016; HEI-2015 scores calculated using the population ratio method with Day 1 dietary intake.

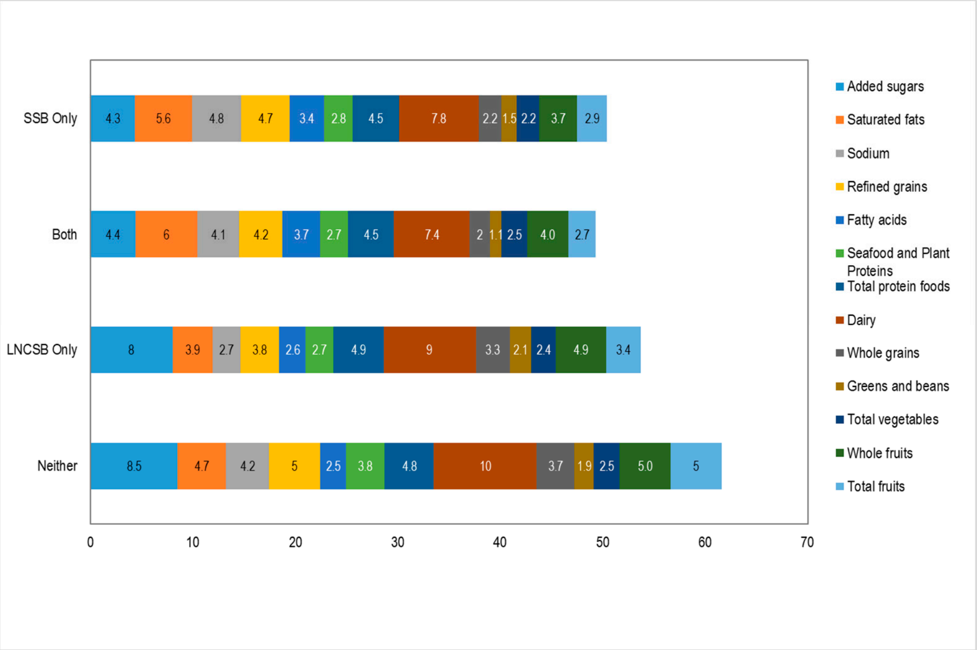

Figure S4. Mean scores for sub-components of the HEI-2015 among children 2-18 years by LNCSB consumer group, NHANES 2009-2016; HEI-2015 scores calculated using the population ratio method with Day 1 dietary intake.

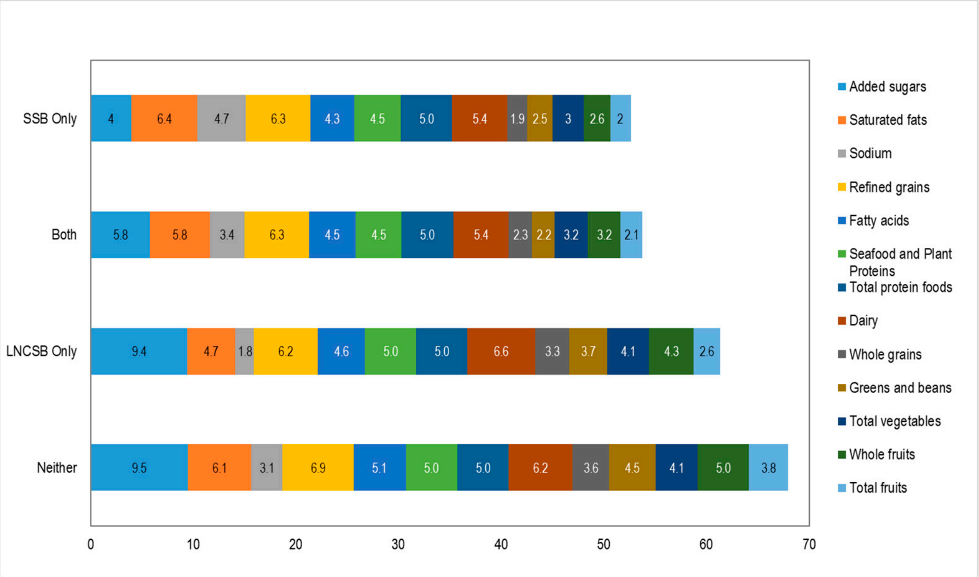

Figure S5. Mean scores for sub-components of the HEI-2015 among adults 19-64 years by LNCSB consumer group, NHANES 2009-2016; HEI-2015 scores calculated using the population ratio method with Day 1 dietary intake.

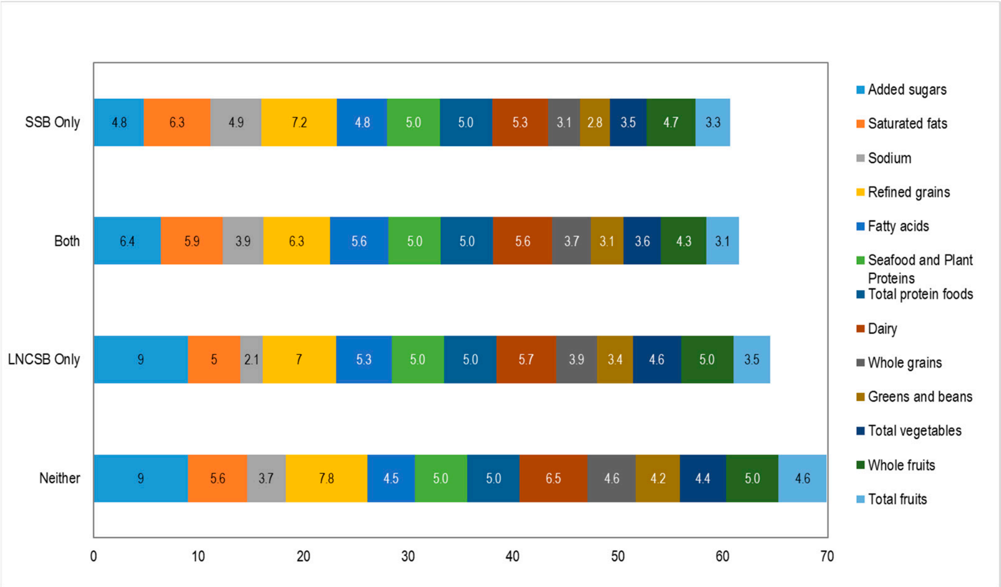

Figure S6. Mean scores for sub-components of the HEI-2015 among older adults 65+ years by LNCSB consumer group, NHANES 2009-2016; HEI-2015 scores calculated using the population ratio method with Day 1 dietary intake.

Table S1. Adjusted HEI-2015 Component scores across Zero-Calorie Unsweetened Beverage (ZCUB) and Sugar-Sweetened Beverage (SSB) consumer groups and lifestages, NHANES 2009-2016.

	SSB Only	Both	ZCUB Only	Neither
	Adjusted relative difference ± standard error (±SE)†			
**2-18 years**				
Total fruits	Ref	1.1 (0.05)*	1.5 (0.07)*	1.6 (0.07)*
Whole fruits	Ref	1.3 (0.06)*	1.6 (0.09)*	1.4 (0.09)*
Total vegetables	Ref	1.1 (0.03)*	1.2 (0.04)*	1.1 (0.06)
Greens and beans	Ref	1.5 (0.16)*	1.8 (0.21)*	1.2 (0.24)
Whole grains	Ref	1.2 (0.08)*	1.7 (0.12)*	1.3 (0.11)*
Dairy	Ref	1.0 (0.02)	1.1 (0.02)*	1.1 (0.04)*
Total protein foods	Ref	1.0 (0.02)	1.1 (0.02)*	1.0 (0.03)
Seafood and Plant Proteins	Ref	1.2 (0.07)*	1.4 (0.09)*	1.3 (0.15)*
Fatty acids	Ref	1.0 (0.03)	0.9 (0.04)*	0.9 (0.07)
Refined grains	Ref	0.9 (0.02)*	0.9 (0.03)*	1.1 (0.05)*
Sodium	Ref	0.9 (0.03)*	0.8 (0.03)*	1.0 (0.04)
Added sugars	Ref	1.3 (0.04)*	2.1 (0.05)*	1.9 (0.07)*
Saturated fats	Ref	0.9 (0.02)*	0.8 (0.02)*	0.8 (0.05)*
**19-64 years**				
Total fruits	Ref	1.4 (0.07)*	1.8 (0.10)*	1.5 (0.15)*
Whole fruits	Ref	1.7 (0.12)*	2.2 (0.16)*	1.6 (0.21)*
Total vegetables	Ref	1.1 (0.03)*	1.2 (0.03)*	1.1 (0.05)
Greens and beans	Ref	1.3 (0.08)*	1.7 (0.10)*	1.1 (0.13)
Whole grains	Ref	1.4 (0.08)*	2.0 (0.11)*	1.4 (0.14)*
Dairy	Ref	1.1 (0.03)*	1.2 (0.03)*	1.1 (0.05)*
Total protein foods	Ref	1.0 (0.02)*	1.1 (0.02)*	1.0 (0.02)
Seafood and Plant Proteins	Ref	1.3 (0.05)*	1.6 (0.06)*	1.2 (0.11)*
Fatty acids	Ref	1.0 (0.03)	1.1 (0.04)*	1.0 (0.06)
Refined grains	Ref	0.9 (0.02)*	0.9 (0.02)*	0.9 (0.04)*
Sodium	Ref	0.8 (0.02)*	0.7 (0.02)*	0.8 (0.05)*
Added sugars	Ref	1.5 (0.05)*	2.6 (0.09)*	2.6 (0.10)*
Saturated fats	Ref	0.9 (0.02)*	0.9 (0.02)*	0.8 (0.05)*
**65+ years**				
Total fruits	Ref	1.1 (0.11)	1.3 (0.13)*	1.0 (0.12)
Whole fruits	Ref	1.2 (0.14)	1.5 (0.17)*	1.1 (0.13)
Total vegetables	Ref	1.1 (0.06)	1.2 (0.07)*	1.1 (0.07)*
Greens and beans	Ref	1.2 (0.18)	1.3 (0.20)	1.3 (0.27)
Whole grains	Ref	1.4 (0.16)*	1.8 (0.18)*	1.7 (0.23)*
Dairy	Ref	1.0 (0.07)	1.1 (0.07)	1.1 (0.10)
Total protein foods	Ref	1.0 (0.03)	1.1 (0.03)	1.0 (0.04)
Seafood and Plant Proteins	Ref	1.1 (0.12)	1.2 (0.13)*	1.0 (0.14)
Fatty acids	Ref	1.0 (0.08)	1.0 (0.07)	1.0 (0.08)
Refined grains	Ref	1.1 (0.05)	1.0 (0.05)	1.1 (0.08)
Sodium	Ref	0.9 (0.05)	0.8 (0.04)*	0.8 (0.07)*
Added sugars	Ref	1.1 (0.07)	1.8 (0.11)*	1.8 (0.12)*
Saturated fats	Ref	1.0 (0.05)	0.9 (0.05)*	0.9 (0.07)

† Linear regression models adjusted for survey cycle, age, sex, race/ethnicity, BMI, household reference education, household poverty income ratio, and dieting status; *Significant at the p=0.05 level.

Table S2. Adjusted HEI-2015 Component scores across Low/No-Calorie Sweetened Beverage (LNCSB) and Sugar-Sweetened Beverage (SSB) consumer groups and lifestages, NHANES 2009-2016.

	SSB Only	Both	LNCSB Only	Neither
	Adjusted relative difference ± standard error(±SE)†			
**2-18 years**				
Total fruits	Ref	1.0 (0.10)	1.1 (0.08)	1.4 (0.05)*
Whole fruits	Ref	1.0 (0.11)	1.3 (0.09)*	1.3 (0.05)*
Total vegetables	Ref	1.1 (0.05)*	1.1 (0.06)	1.1 (0.03)*
Greens and beans	Ref	0.7 (0.16)	1.0 (0.16)	1.3 (0.09)*
Whole grains	Ref	0.8 (0.10)	1.3 (0.12)*	1.5 (0.06)*
Dairy	Ref	0.9 (0.04)	1.1 (0.03)	1.1 (0.02)*
Total protein foods	Ref	1.0 (0.06)	1.1 (0.03)*	1.0 (0.01)
Seafood and Plant Proteins	Ref	1.0 (0.12)	1.1 (0.11)	1.2 (0.05)*
Fatty acids	Ref	1.1 (0.08)	1.0 (0.07)	0.9 (0.03)*
Refined grains	Ref	0.9 (0.06)	0.9 (0.05)*	1.0 (0.02)
Sodium	Ref	0.9 (0.05)	0.7 (0.05)*	0.9 (0.02)*
Added sugars	Ref	1.0 (0.06)	1.7 (0.05)*	1.7 (0.03)*
Saturated fats	Ref	1.0 (0.04)	0.8 (0.04)*	0.9 (0.02)*
**19-64 years**				
Total fruits	Ref	1.1 (0.08)	1.1 (0.06)	1.4 (0.05)*
Whole fruits	Ref	1.1 (0.09)	1.2 (0.06)*	1.5 (0.05)*
Total vegetables	Ref	1.0 (0.03)	1.1 (0.02)*	1.1 (0.02)*
Greens and beans	Ref	0.9 (0.09)	1.2 (0.06)*	1.3 (0.05)*
Whole grains	Ref	1.1 (0.09)	1.4 (0.07)*	1.6 (0.06)*
Dairy	Ref	1.0 (0.04)	1.1 (0.03)*	1.1 (0.02)*
Total protein foods	Ref	1.0 (0.02)*	1.1 (0.01)*	1.0 (0.01)*
Seafood and Plant Proteins	Ref	1.0 (0.06)	1.1 (0.04)*	1.3 (0.04)*
Fatty acids	Ref	1.0 (0.05)	1.0 (0.03)	1.1 (0.02)*
Refined grains	Ref	0.9 (0.03)	0.9 (0.02)*	1.0 (0.02)
Sodium	Ref	0.8 (0.05)*	0.6 (0.03)*	0.8 (0.02)*
Added sugars	Ref	1.2 (0.04)*	1.9 (0.04)*	1.9 (0.03)*
Saturated fats	Ref	0.9 (0.03)*	0.8 (0.02)*	1.0 (0.02)*
**65+ years**				
Total fruits	Ref	1.1 (0.10)	1.0 (0.05)	1.2 (0.05)*
Whole fruits	Ref	1.0 (0.11)	1.1 (0.06)*	1.3 (0.06)*
Total vegetables	Ref	1.0 (0.05)	1.1 (0.03)*	1.1 (0.03)*
Greens and beans	Ref	1.2 (0.17)	1.1 (0.11)	1.2 (0.10)*
Whole grains	Ref	1.2 (0.11)	1.2 (0.11)*	1.3 (0.07)*
Dairy	Ref	1.1 (0.09)	1.0 (0.05)	1.2 (0.04)*
Total protein foods	Ref	1.1 (0.03)*	1.1 (0.02)*	1.0 (0.02)*
Seafood and Plant Proteins	Ref	1.3 (0.12)*	1.1 (0.07)	1.2 (0.05)*
Fatty acids	Ref	1.1 (0.08)	1.1 (0.04)	1.0 (0.03)
Refined grains	Ref	0.9 (0.05)	0.9 (0.03)	1.0 (0.03)
Sodium	Ref	0.9 (0.06)	0.6 (0.04)*	0.9 (0.03)*
Added sugars	Ref	1.2 (0.05)*	1.7 (0.05)*	1.7 (0.04)*
Saturated fats	Ref	1.0 (0.05)	0.9 (0.03)*	0.9 (0.03)*

† Linear regression models adjusted for survey cycle, age, sex, race/ethnicity, BMI, household reference education, household poverty income ratio, and dieting status; *Significant at the *p*=0.05 level.
